# Changes in Serum MicroRNAs after Anti-IL-5 Biological Treatment of Severe Asthma

**DOI:** 10.3390/ijms22073558

**Published:** 2021-03-30

**Authors:** Manuel J. Rial, José A. Cañas, José M. Rodrigo-Muñoz, Marcela Valverde-Monge, Beatriz Sastre, Joaquín Sastre, Victoria del Pozo

**Affiliations:** 1 Allergy Unit, Hospital Universitario Fundación Jiménez Díaz, 28040 Madrid, Spain; manuterial@gmail.com (M.J.R.); marcela.valverde@quironsalud.es (M.V.-M.); JSastre@fjd.es (J.S.); 2 Department of Immunology, IIS-Fundación Jiménez Díaz, 28040 Madrid, Spain; toniosego@msn.com (J.A.C.); jose.rodrigom@quironsalud.es (J.M.R.-M.); bssastre@fjd.es (B.S.); 3 CIBER de Enfermedades Respiratorias (CIBERES), Instituto de Salud Carlos III, 28029 Madrid, Spain

**Keywords:** severe asthma, biomarkers, microRNAs, anti-IL5 biologics, mepolizumab, reslizumab

## Abstract

There is currently enough evidence to think that miRNAs play a role in several key points in asthma, including diagnosis, severity of the disease, and response to treatment. Cells release different types of lipid double-membrane vesicles into the extracellular microenvironment, including exosomes, which function as very important elements in intercellular communication. They are capable of distributing genetic material, mRNA, mitochondrial DNA, and microRNAs (miRNAs). Serum miRNA screening was performed in order to analyze possible changes in serum miRNAs in 10 patients treated with reslizumab and 6 patients with mepolizumab after 8 weeks of treatment. The expression of miR-338-3p was altered after treatment (*p* < 0.05), although no significant differences between reslizumab and mepolizumab were found. Bioinformatic analysis showed that miR-338-3p regulates important pathways in asthma, such as the MAPK and TGF-β signaling pathways and the biosynthesis/degradation of glucans (*p* < 0.05). However, it did not correlate with an improvement in lung function. MiRNA-338-3p could be used as a biomarker of early response to reslizumab and mepolizumab in severe eosinophilic asthmatic patients. In fact, this miRNA could be involved in airway remodeling, targeting genes related to MAPK and TGF-β signaling pathways.

## 1. Introduction

Airway inflammation in asthma is distinguished by an imbalance between T1/T2 immune responses. T-helper 2 (Th2) cells produce interleukin (IL)-4, IL-5, IL-6, and IL-13, which are responsible for the allergic immune response [1]. A consequence of the upregulation of T2 cytokines is airway eosinophilic inflammation and the subsequent manifestations of asthma. Eosinophils participate in the initiation phase towards Th2 polarization, in the suppression of the Th1/Th17 pathways in the pulmonary lymph nodes, in the recruitment of Th2 cells in the lung, and in the mechanisms of resolution of inflammation that restore pulmonary homeostasis [2]. All these characteristics of eosinophils imply that they play an important role in asthma, not only as a biomarker of severity and/or control of asthma symptoms but also as a regulator of multiple functions. In fact, there is currently an important therapeutic development underway with biologics, whose target is the eosinophil directly (anti-IL5Rα: benralizumab) [3] or indirectly (anti-IL-5: mepolizumab and reslizumab) [4,5]. Additionally, eosinophils can release exosomes containing specific and nonspecific proteins as well as microRNAs (miRNAs); those have recently gained importance as regulatory elements that can be transferred to recipient cells. To date, asthmatic patients have been identified to have differential expression of more than 100 miRNAs when compared with healthy subjects [6].

MiRNAs are short RNA sequences (around 19–22 nucleotides in length) with crucial functions in post-transcriptional regulation. They are involved in the development and continuation of pathogenic mechanisms of several diseases, including asthma [7]. One of the unique characteristics of miRNAs is that they are resistant to degradation by nucleases, making them very promising and stable biomarkers. It is possible that asthmatic inflammation may be maintained as a consequence of altered miRNA expression profiles, which regulate some of the complex inflammatory processes that occur in asthma [8]. All these characteristics make miRNAs an emerging field in allergic asthma, specifically as possible biomarkers. For clinical application, the most important criteria for a diagnostic and prognostic biomarker should be high sensitivity and specificity. However, there are several limitations that hinder the use of miRNAs in clinical practice, such as the lack of studies with a large sample size, the criteria used in clinical applications (like age and gender), and the methods used (such as equipment, techniques or qualified staff) [9]. Regarding current clinical practice, there is a lack of standardized protocols on the use of miRNAs; however, there is promising evidence to believe that they constitute a useful tool for future use [10,11]. For example, our research group has demonstrated that serum miRNAs can differentiate between different asthma severities grades [12]. Likewise, a study made by our lab group has recently described that miRNAs can be used as a useful tool to predict early response to benralizumab in severe eosinophilic asthmatics [13].

The usefulness of miRNAs as markers of the therapeutic response to inhaled treatment has been previously explored [14]; however, the change in these miRNAs, secondary to biological treatment, has been poorly addressed. The purpose of this study is to analyze possible changes in serum miRNAs in patients treated with reslizumab and mepolizumab after 8 weeks of treatment. Both treatments are humanized immunoglobulin G (IgG) monoclonal antibodies with a high affinity for IL-5, neutralizing this cytokine by binding to epitopes on the IL-5-Rα binding domain.

## 2. Results

### 2.1. Administration of Anti-IL-5 Drugs Improves Asthma Symptoms

To develop this study, a total of 16 medicated severe eosinophilic patients with anti-IL-5 drugs were included; 6 were treated with mepolizumab and 10 with reslizumab. Clinical and demographic characteristics before the first administration of biologicals are shown in Table 1.

The studied population consisted of adult patients (58 ± 13 years), and mostly, they were women (68.75%). Regarding inflammatory characteristics, more than half of the recruited patients were atopic, and a high mean of immunoglobulin E (IgE) was observed (Table 1). Additionally, this asthmatic population showed peripheral blood eosinophil counts higher than normal reference levels as well as raised fractional exhaled nitric oxide (FeNO) levels (Table 1).

**Figure 1:**
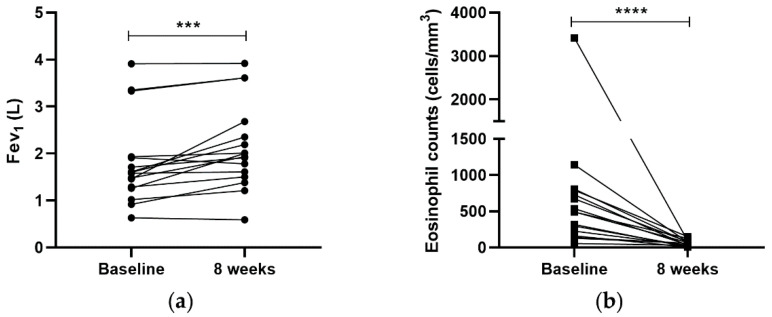
Anti-IL-5 biologics improved lung function and decreased peripheral eosinophils counts. Lung function (**a**) and peripheral eosinophil levels (**b**) were measured at baseline and an 8-week follow-up visit. After 8 weeks of mepolizumab or reslizumab drug administration, patients recovered their FEV_1_ and lessened their peripheral eosinophils counts. *** *p* < 0.001, **** *p* < 0.0001.

After 8 weeks of biological treatment, 87.5% of the patients improved their lung function in a significant way (1.81 ± 0.93 vs. 2.14 ± 0.92 L; *p* < 0.001). Additionally, with respect to peripheral blood eosinophils, all asthmatic patients presented a decrease in peripheral eosinophil counts, reaching normal levels. This reduction was very significant (649.86 ± 798.19 vs. 58.81 ± 40.49 cells/mm^3^; *p* < 0.0001), corroborating that biological drugs were able to decrease the peripheral blood eosinophils (Figure 1).

In view of these data, mepolizumab and reslizumab are able to improve clinical parameters from severe asthmatics patients in a time-point of 8 weeks.

### 2.2. MiRNA Deregulation after Anti-IL5 Treatment

**Figure 2:**
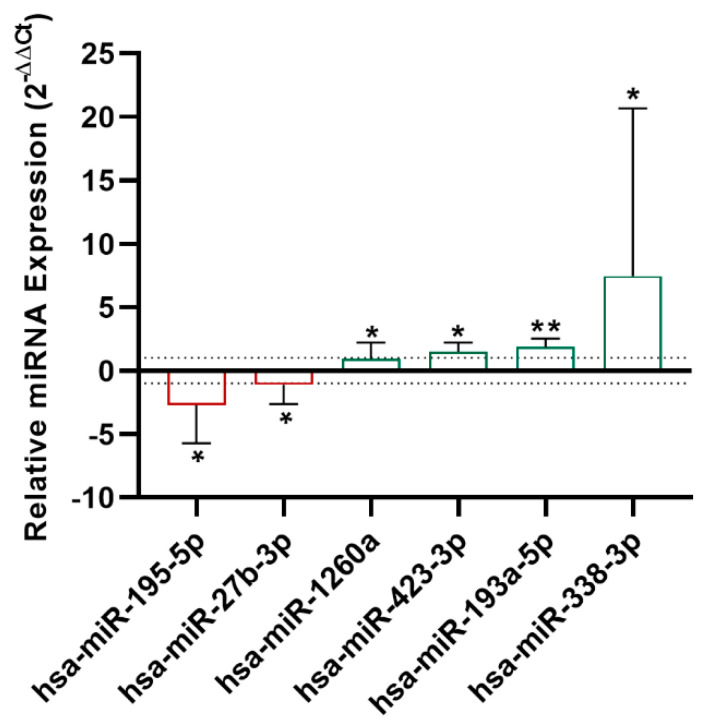
Serum miRNA deregulation in severe asthmatic patients treated with anti-IL-5 drugs. Among the nine patients analyzed, five were severe asthmatics treated with mepolizumab and four with reslizumab. Eosinophilic asthmatic patients showed an altered expression of miR-195-5p, miR-27b-3p, miR-1260a, miR-423-3p, miR-193a-5p, and miR-338-3p at eight weeks after anti-IL-5 administration. Relative miRNA expression is expressed as 2^−ΔΔCt^. * *p* < 0.05, ** *p* < 0.01.

A miRNA screening was performed with serum samples from nine treated asthmatic patients using the miRNA PCR array, evaluating 179 miRNAs. Results from the miRNA array showed that miR-195-5p and miR-27b-3p were downregulated (*p* < 0.05), while miR-1260a (*p* < 0.05), miR-193a-5p (*p* < 0.01), and miR-338-3p (*p* < 0.05) were upregulated at 8 weeks (Figure 2).

**Figure 3:**
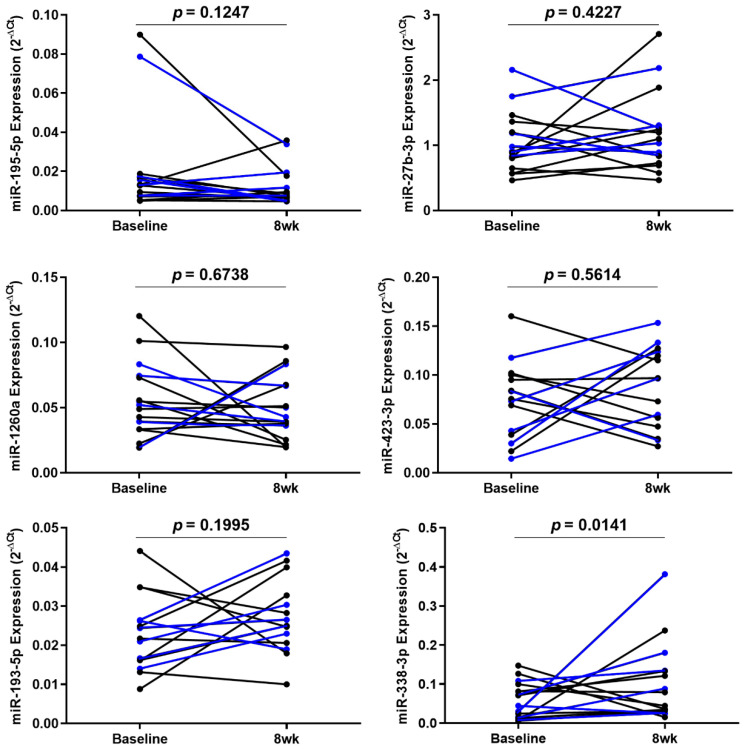
Individual variation of miR-195-5p, miR-27b-3p, miR-1260a, miR-423-3p, miR-193a-5p, and miR-338-3p after 8 weeks of treatment. Relative expressions of these miRNAs were validated by RT-qPCR in serum samples from 16 asthmatic patients treated with mepolizumab or reslizumab. Among these miRNAs, miR-338-3p was the only one that modified its expression in a significant way. All experiments were performed in triplicate. Relative miRNA expression is expressed as 2^−ΔCt^. Blue dots and lines represent patients treated with mepolizumab. Black dots and lines represent patients treated with reslizumab.

Afterward, these results were validated by RT-qPCR of serum samples from 16 severe asthmatics. Nine of them were the same patients that we used in the miRNA PCR array, and the rest were different patients treated with mepolizumab or reslizumab. We confirmed that only miR-338-3p was significantly upregulated (*p* < 0.05) in these patients after 8 weeks of treatment (Figure 3).

In view of these results, we tried to study the effect of mepolizumab and reslizumab separately. However, no statistical differences were found when miRNA expression was compared between the mepolizumab groups and the reslizumab group (data not shown).

### 2.3. MiR-338-3p Regulates Important Pathways in Asthma but It Does Not Correlate with Clinical Parameters

Afterward, we performed an in-silico analysis with the bioinformatic tool DIANA-mirPath to obtain the putative target genes of miR-338-3p and the altered pathways. In this analysis, an alteration in some target genes and pathways related to several functions and processes in asthma were observed, such as mitogen-activated protein kinases (MAPK) and transforming growth factor beta (TGF-β) signaling pathways and glycan biosynthesis/degradation (Table 2).

On the other hand, we also studied whether any of the miRNAs were correlated with circulating eosinophils at 8 weeks. Although a mild negative correlation was observed between ΔCt values of miR-338-3p and eosinophil counts, no significant differences were reached (r = −0.2426, *p* > 0.05).

We also evaluated whether the levels of miR-338-3p correlated with lung function in forced expiratory volume in 1 s (FEV_1_) terms, but no significant differences were found (r = 0.138; *p* > 0.05).

## 3. Discussion

In this study, we described that miRNA-338-3p could be used as a biomarker of early anti-IL5 biologic response (reslizumab and mepolizumab) in severe eosinophilic asthmatic patients. The usefulness of miRNAs as markers of the therapeutic response to inhaled treatment in asthma has been previously explored [14]; however, the variation in miRNAs due to biological treatment has only been analyzed after benralizumab treatment [13]; it has never been addressed with anti-IL5 drugs such as mepolizumab and reslizumab.

We observed that miRNA-338-3p is upregulated in serum from severe asthmatic patients after 8 weeks of anti-IL-5 treatment. This data concurs with results obtained previously [13]. In this report, our group showed that three miRNAs were altered after 8 weeks of benralizumab administration, and miR-338-3p was one of them.

MiR-338 has been described to regulate differentiation, apoptosis, and probably tissue degeneration [15]. Other authors have speculated that inflammation and cell proliferation at the base of the remodeling processes can be promoted by the activation of miR-338 [16]. However, the role of miRNA-338 in lung functions has not been clearly identified, but the findings to date appear to implicate it in the pathogenesis of obstructive lung diseases [15,16].

In view of these results, we could speculate that changes in the expression of miR-338-3p could translate into significant changes in lung function. In most of the patients recruited, a great improvement in FEV_1_ was observed 8 weeks after the introduction of the anti-IL-5 drug (FEV_1_ baseline 1.81 ± 0.93 L; FEV_1_ 8 weeks 2.14 ± 0.92 L); however, this improvement in lung function seems to behave independently of the variation of miR-338 at 8 weeks in this population, as correlation results have shown. One possible explanation is that this fact may be due to the small sample size used, which is one of the main limitations of the study. On the other hand, it could be related to the fact that FEV_1_ is not the best parameter that translates the miR-338-3p clinical expression, nor is it the parameter that changes the most with treatment, compared to other parameters such as quality of life or reduction of oral corticosteroids. So, it would be very interesting to be able to find a biomarker that could predict a good clinical response to a biological drug in a short period of time since this would save time and money for healthcare providers in the field of severe asthma. However, currently, there is a lack of standardized protocols about the use of miRNAs in clinical practice [10,11]; hence, further studies are needed.

Despite the important findings of this study, there are some limitations. First, this study was performed with a small sample size, although the results and differences of miR-338-3p expression between the groups were found to be significant. Second, we have included a unique population treated with anti-IL-5 biologics (reslizumab or mepolizumab); it would be interesting to study the effects of miRNA expression of reslizumab and mepolizumab separately. Hence, more studies are necessary to confirm this finding on a larger scale.

To our knowledge, this study supports the idea that there are differences in the expression of certain miRNAs after the introduction of an anti-IL-5 biological treatment. The expression of miR-338-3p changed after treatment; therefore, this could be used as an early response biomarker to anti-IL-5 biological drugs. However, more studies are needed to relate these changes to any clinical improvement observed after 8 weeks of treatment with an anti-IL-5 drug in patients with severe asthma.

## 4. Materials and Methods

### 4.1. Patient Selection

Sixteen severe eosinophilic asthmatic patients treated with anti-IL-5 drugs were recruited from Hospital Universitario Fundación Jiménez Díaz in Madrid. Six of them were treated with mepolizumab and the rest with reslizumab. Asthma diagnosis and treatment were made according to the Global Initiative for Asthma (GINA) guidelines [17]. The patient inclusion criterion for the administration of mepolizumab and reslizumab was the presence of more than 300 and 400 eosinophils/µL in peripheral blood (in at least one registry in the previous year), respectively. The electronic medical record included pulmonary function, blood count, ACT, medication and visits to the emergency room. However, not all data were available in the electronic registry for all patients, so some parameters could not be included for analysis.

Patients received all necessary information, and they signed a form of written informed consent to participate. The study was conducted following the principles of the Declaration of Helsinki and approved by Fundación Jiménez Díaz Ethics Committee.

### 4.2. Blood Processing

Peripheral blood samples were collected in anticoagulant-free tubes (Becton Dickinson, Franklin Lakes, NJ, USA). Serum samples were obtained by blood clotting and centrifugation at 3000 rpm for 10 min at 4 °C. Then, they were stored at −80 °C until use.

### 4.3. MiRNAs Isolation

MiRNAs were obtained from 200 μL of serum using a miRNeasy serum/plasma advanced kit (Qiagen, Hilden, Germany), according to the manufacturer’s instructions. Three synthetic miRNA spike-ins (SP2, SP4, and SP5) were added to evaluate optimal RNA extraction (miRCURY LNA RNA Spike-in kit, Qiagen, Hilden, Germany).

### 4.4. cDNA Retrotranscription

Serum miRNAs were retrotranscribed to cDNA using the miRCURY LNA RT Kit (Qiagen, Hilden, Germany), following the manufacturer’s protocol. Briefly, 2 μL of total RNA was mixed with reverse transcription enzyme and with another synthetic miRNA Spike-in (Sp6) and cel-miR-39-3p, which accounts for control of a correct retrotranscription to cDNA, yielding a total volume of 10 μL. The reaction was performed for 60 min at 42 °C, then 5 min at 95 °C, and immediately at 4 °C in a Veriti Thermal Cycler (Applied Biosystems, Warrington, UK).

### 4.5. Serum miRNA PCR Panel

In order to perform an initial serum miRNA screening of the asthmatic patients, Serum/Plasma miRNA PCR Panels (Qiagen, Hilden, Germany) to evaluate 179 miRNAs were used. Additionally, the PCR panels contained several controls for RNA isolation (UniSp2, UniSp4, and UniSp5); they also monitored cDNA synthesis (UniSp6 and cel-miR-39-3p) and checked that reaction was successful (UniSp3). Additionally, these panels included miR-451 and miR-23a, which were used as hemolysis markers. Moreover, miR-191-5p, miR-let-7a-5p, and cel-miR-39-3p were utilized as endogenous controls for data normalization. MiRNA expression was calculated by using the 2^−ΔΔCt^ method [18], where ΔCt = Ct_miRNA_ − $\bar{X}$ (Ct_miR-191-5p_ + Ct_let-7a-5p_ + C_tcel-miR-39-3p_) and ΔΔCt = ΔCt_8wk_ − ΔCt_Baseline_.

Nine serum samples from severe asthmatics were analyzed; five of them were treated with mepolizumab and four with reslizumab. Immediately after cDNA synthesis, it was diluted 1:30 in RNase-free water, and the reaction was performed in a Light Cycler^®^ 96 thermocycler (Roche, Basel, Switzerland). The incubation program was carried out for 45 cycles of 95 °C during 10 s and 60 °C for 1 min. DNA melting was performed by heating at 95 °C for 5 s, then 65 °C for 1 min, and finally at 97 °C for 1 s. Samples were cooled for 10 s at 40 °C. Cycle threshold (Ct) data were taken in the last step of the melting cycle in a continuous way.

### 4.6. MiRNA Validation

MiRNAs were validated in 16 patients, including the 9 patients used in the initial screening. Validation was performed by RT-qPCR using a miRCURY LNA SYBR Green PCR Kit (Qiagen, Hilden, Germany) according to the manufacturer’s instructions. Briefly, cDNA from serum was diluted 1:30 in RNase-free water and then mixed with SYBR Green and the suitable miRNA probes (miR-195-5p, miR-27b-3p, miR-1260a, miR-423-3p, miR193a-3p, and miR-338-3p) (Qiagen, Hilden, Germany). Additionally, miR-191-5p, let-7a-5p, and cel-miR-39 were used as endogenous controls (Qiagen, Hilden, Germany). MiR-451a and miR-23a-3p were utilized for hemolysis control. All samples were run in triplicate, and the reaction was performed in a Light Cycler^®^ 96 thermocycler (Roche, Basel, Switzerland); cycle threshold (Ct) values were analyzed with LightCycler^®^ 96 SW 1.1 (Roche, Basel, Switzerland) software. MiRNA expression was calculated by using the 2^−ΔCt^ method [18], where ΔCt = Ct_miRNA_ − (Ct_miR-191-5p_ + Ct_let-7a-5p_ + C_tcel-miR-39-3p_).

### 4.7. Analysis of Pathway Enrichment

In order to find the specific pathways and biological functions that miR-338-3p is involved in, an in-silico study of enrichment analysis with this miRNA was performed by using DIANA-mirPath v3 (DIANA LAB, University of Thessaly, Thessaly, Greece) and the DIANA-TarBase database v7.0 (DIANA LAB, University of Thessaly, Thessaly, Greece) [19]. We report KEGG pathways with a *p*-value and a false discovery rate (FDR) of less than 0.05.

### 4.8. Statistical Analysis

A comparison between groups was performed using the paired two-tailed *t*-test for parametric data, with the Wilcoxon matched-pair test for non-Gaussian data. Unpaired groups were compared by a two-tailed Student *t*-test for Gaussian parameters and the Mann–Whitney U-test for non-Gaussian samples. Normality was analyzed using the Shapiro–Wilk test. For the analysis of categorical variables, Fisher’s exact test was used. A *p*-value < 0.05 was considered significant. Statistical calculations and graphs were performed using GraphPad Prism 8.4 (GraphPad Software Inc., San Diego, CA, USA).

## 5. Conclusions

In this study, we report evidence supporting the potential use of miR-338-3p as a biomarker of an early anti-IL5 biologic response in severe eosinophilic asthmatic patients. In fact, this miRNA could be involved in airway remodeling, targeting genes related to MAPK and TGF-β signaling pathways.

## Figures and Tables

**Table 1:** Initial demographic and clinical characteristics of the 16 patients.

Demographic and Clinical Characteristics		
Demographic	Age ^1^	58 ± 13
	Female (%)	11 (68.75)
	Age at onset	
	<30 years (%)	35.7
	>30 years (%)	64.3
	Body Mass Index ^1^	26.90 ± 5.29
	Smoking status	
	Never (%)	62.5
	Passive (%)	6.25
	Former smoker (%)	25
	Smoker (%)	6.25
Inflammatory characteristics	Atopy (%)	53.8
	Total IgE ^1^ (kU/L)	603.7 ± 663.3
	Eosinophils (cells/mm^3^)^1^	493 ± 321
	FeNO ^1^ (ppb)	56.08 ± 38.1
Functional parameters	FEV_1_ Pre-BD (%) ^1^	74.69 ± 29.21
	FEV_1_ Post-BD (%) ^1^	80.25 ± 31.98
	FVC Pre-BD (%) ^1^	86.87 ± 20.24
	FVC Post-BD (%) ^1^	87.62 ± 38.85
	FEV_1_/FVC Pre-BD ^1^	69.13 ± 11.64
	FEV_1_/FVC Post-BD ^1^	70.2 ± 9.73
Questionaries	ACT ^1^	13.77 ± 6.2

^1^ Results are expressed as mean ± SD.

**Table 2:** KEGG pathways significantly altered by miR-338-3p.

KEGG Pathway	*p*-Value	Target Genes
Prion diseases	2.69^−31^	*PRNP*
Fatty acid biosynthesis	2.42^−29^	*FASN*
Fatty acid metabolism	5.22^−7^	*FASN*
Other types of O-glycan biosynthesis	1.49^−6^	*OGT, POMT2, EOGT, POFUT1*
MAPK signaling pathway	0.015	*FOS, CACNG8, DUSP2, ELK4, CDC25B, TAOK2, MAP4K3, MAP2K3, RASA1, ZAK, RAPGEF2, NFKB2, MAPKAP3, HSPA8, CACNA1H, MAP3K2, DUSP5, RPSKA4, NFATC3, DUSP1*
Other glycan degradation	0.026	*NEU3*
TGF-beta signaling pathway	0.029	*SKP1, DCN, SMAD4, SMAD5, SP1, BAMBI*
Glutathione metabolism	0.031	*SRM, CDC1, GSTP1, GGT6, RRM1*
Cell cycle	0.032	*YWHAH, CCNB1, CDC25B, MCM4, BUB3, SKP1, CCND1, SMAD4, CDC14B, PRKDC, MDM2, MCM3, CDC25A*
Mucin type O-Glycan biosynthesis	0.036	*GALNT7, GALNT16*

## Data Availability

The data presented in this study are available on request from the corresponding author. The data are not publicly available due to it includes personal data of patients.
